# Mitogenomic phylogenetic analyses of the Delphinidae with an emphasis on the Globicephalinae

**DOI:** 10.1186/1471-2148-11-65

**Published:** 2011-03-10

**Authors:** Julia T Vilstrup, Simon YW Ho, Andrew D Foote, Phillip A Morin, Danielle Kreb, Michael Krützen, Guido J Parra, Kelly M Robertson, Renaud de Stephanis, Philippe Verborgh, Eske Willerslev, Ludovic Orlando, M Thomas P Gilbert

**Affiliations:** 1 Centre for GeoGenetics, Natural History Museum of Denmark, University of Copenhagen, Øster Voldgade 5-7, 1350 Copenhagen, Denmark; 2 School of Biological Sciences, University of Sydney, Sydney NSW 2006, Australia; 3 Southwest Fisheries Science Center, NOAA Fisheries, 3333 N. Torrey Pines Ct., La Jolla, CA, 92037 USA; 4 Yayasan Konservasi RASI, Samarinda, Kalimantan Timur, Indonesia; 5 Evolutionary Genetics Group, Anthropological Institute and Museum, University of Zurich, Winterthurerstr. 190, 8057 Zurich, Switzerland; 6 School of Biological Sciences, Flinders University, Lincoln Marine Science Centre, GPO BOX 2100 Adelaide, SA 5001, Australia; 7 South Australian Research and Development Institute (Aquatic Sciences), PO Box 120, Henley Beach, SA 5022, Australia; 8 CIRCE, Conservation Information and Research on Cetaceans, C/Cabeza de Manzaneda 3, Algeciras-Pelayo, 11390 Cadiz, Spain; 9 Departamento de Biologia de la Conservación, Estación Biologica de Donana, CSIC, C/Americo Vespucio S/N, Isla de la Cartuja, Sevilla, 41092, Spain

## Abstract

**Background:**

Previous DNA-based phylogenetic studies of the Delphinidae family suggest it has undergone rapid diversification, as characterised by unresolved and poorly supported taxonomic relationships (polytomies) for some of the species within this group. Using an increased amount of sequence data we test between alternative hypotheses of soft polytomies caused by rapid speciation, slow evolutionary rate and/or insufficient sequence data, and hard polytomies caused by simultaneous speciation within this family. Combining the mitogenome sequences of five new and 12 previously published species within the Delphinidae, we used Bayesian and maximum-likelihood methods to estimate the phylogeny from partitioned and unpartitioned mitogenome sequences. Further *ad hoc *tests were then conducted to estimate the support for alternative topologies.

**Results:**

We found high support for all the relationships within our reconstructed phylogenies, and topologies were consistent between the Bayesian and maximum-likelihood trees inferred from partitioned and unpartitioned data. Resolved relationships included the placement of the killer whale (*Orcinus orca*) as sister taxon to the rest of the Globicephalinae subfamily, placement of the Risso's dolphin (*Grampus griseus*) within the Globicephalinae subfamily, removal of the white-beaked dolphin (*Lagenorhynchus albirostris*) from the Delphininae subfamily and the placement of the rough-toothed dolphin (*Steno bredanensis*) as sister taxon to the rest of the Delphininae subfamily rather than within the Globicephalinae subfamily. The additional testing of alternative topologies allowed us to reject all other putative relationships, with the exception that we were unable to reject the hypothesis that the relationship between *L. albirostris *and the Globicephalinae and Delphininae subfamilies was polytomic.

**Conclusion:**

Despite their rapid diversification, the increased sequence data yielded by mitogenomes enables the resolution of a strongly supported, bifurcating phylogeny, and a chronology of the divergences within the Delphinidae family. This highlights the benefits and potential application of large mitogenome datasets to resolve long-standing phylogenetic uncertainties.

## Background

The mitochondrial genome is typically non-recombining, has a relatively high substitution rate, and has a smaller effective population size than the nuclear genome [1]. These properties can increase the probability of congruence between the mitochondrial gene tree and the species tree, helping to resolve relationships between recently divergent taxa [2]. Under some conditions, however, phylogenetic analyses of mitochondrial DNA (mtDNA) sequence data can fail to resolve the relationships among taxa into a fully bifurcating tree. Theoretical and empirical studies suggest that greater phylogenetic resolution and bootstrap support for inter-specific nodes should be achievable by increasing the amount of sequence data [3-7]. Additionally, including sequence data from more than one gene will reduce the influence of any variation between genes in phylogenetic signal due to selection or the effects of stochastic lineage sorting [8].

However, not all polytomic relationships are 'soft', and some cannot be resolved by adding sequence data [9]. In some cases even complete mitochondrial genomes have failed to resolve multifurcating relationships (e.g., [10]). Although unlikely [11], a 'hard' molecular polytomy could be the result of a true simultaneous speciation event into multiple daughter species [9,12-14]. Such events could occur when multiple populations of an ancestral species become simultaneously isolated during periods of rapid environmental change [12]. Simultaneous adaptive radiation into multiple species could also occur in sympatry due to intra-specific competition and assortative mating if species occupy a narrow niche width [15].

The use of high-throughput sequencing and pooled tagging methods [16] has the potential to generate large amounts of manifold-coverage sequence data for large numbers of samples quickly and at a relatively low cost, allowing improved resolution of phylogenetic relationships to be achieved routinely (e.g., [17]). Such an approach can allow the differentiation between rapid and simultaneous cladogenesis and, when combined with additional tests, can determine if a molecular polytomy reflects a true species polytomy (e.g., [18-20]).

Previous phylogenetic studies using a variety of markers have suggested an episodic rapid rate of speciation within the Delphinidae family [21-28]. This has resulted in several long-standing uncertainties in the phylogenetic relationships within this group. Analysis of sequences of the cytochrome *b *gene (1,140 bp) [21] led to a number of suggested taxonomic revisions from previous classifications based on morphology [29]. However, a number of these revisions had poor support and required further analysis, e.g., the inclusion of *Grampus griseus *(Risso's dolphin), and removal of *Orcinus orca *(killer whale) from the Globicephalinae subfamily, and the grouping of *O. orca *with the genus *Orcaella *into a proposed Orcininae subfamily. The genus *Lagenorhynchus *was found to be polyphyletic, with *L. albirostris *and *L. acutus *removed from the remaining four congeners and not found to be closely related to each other [21]. There was high support for paraphyly of the genera *Tursiops *and *Stenella *[21], which has been subsequently strengthened by mitogenomic and multi-locus analyses [25,28]. More recent studies, which included both mtDNA and nuclear DNA (nuDNA), suggested an additional revision of the placement of *Steno bredanensis *(rough-toothed dolphin) with *Orcaella *[24,26]; however, another multi-locus study grouped *S. bredanensis *with the *Sotalia *genus, consistent with previous classifications [27]. These more recent analyses using both nuDNA and mtDNA [24,26,27] have failed to confirm all of the revisions suggested by LeDuc *et al*. [21] and have not produced consistent estimates of relationships within this family. For example the ordering of the branches containing the species *O. orca *and *L. albirostris*, and the subfamilies Delphininae, Globicephilinae, and Lissodelphininae has differed considerably among studies [24,26,27]. In addition to being highly variable, the placement of *L. albirostris *has typically been one of the most weakly supported [21,26,27].

Here we estimate the phylogeny of Delphinidae using complete mitogenomes generated using high-throughput sequencing. This is the first time complete mitogenome sequences have been published for five of the species, and mitogenome sequences from a further three species have previously only been used as outgroup species for an intra-specific phylogenetic study on the killer whale [17]. In total, mitogenome sequences from 17 of the 37 extant species within Delphinidae were included in the analyses. This case study on a rapidly radiating group is one of the first to test the power of large sequence datasets produced by parallel-tagged high-throughput sequencing in combination with improved analytical techniques to address long-standing phylogenetic uncertainties. Specifically we test three revisions suggested by LeDuc *et al*. [21] regarding the placement of *O. orca*, *G. griseus*, and *L. albirostris*, and a further suggested revision regarding the placement of *S. bredanensis *[24]. We ultimately test the hypothesis that these uncertainties result from a true species polytomy caused by simultaneous speciation.

## Results and Discussion

### Sequencing

**Figure 1:**
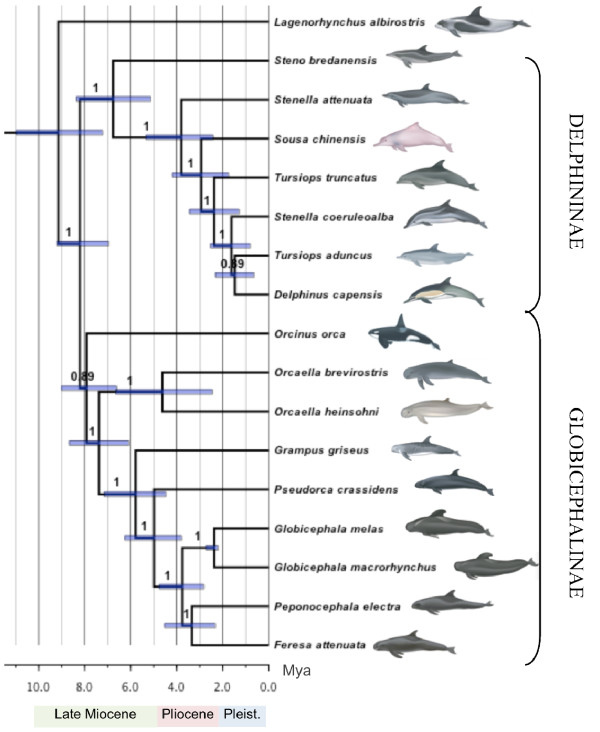
**Bayesian phylogenetic reconstruction of selected taxa within Delphinidae based on analysis of 21 partitioned mitogenome sequences under the uncorrelated lognormal relaxed clock model**. Node labels indicate posterior probabilities and node bars the 95% HPD of the estimated node age. Outgroup taxa used to root the tree include *L. vexillifer, I. geoffrensis, M. monoceros*, and *P. phocoena*, and are not shown. Illustrations are provided by U. Gorter, (not to scale).

In total 18 mitogenome sequences were generated for this study, including multiple representatives per species. Eight of these sequences were incomplete (spanning between 10,681 and 16,672 bp), Table 1; Additional file 1), however at least 1 complete genome was sequenced for each species. The 10 complete sequences had on average 20× coverage of the whole mitogenome of approximately 16,445 bp. In combination with previously published mtDNA genome sequences we were able to use this dataset to reconstruct the most complete and highly resolved mitogenome phylogeny of Delphinidae to date (Figure 1).

**Table 1:** Samples used in study, including outgroup and Delphininae sequences from Genbank (accession numbers are given), and multiple specimens per species used in some analyses.

Species	Common name	**Genbank Acc. No**.	Source
*Lagenorhynchus albirostris*	White beaked dolphin	AJ554061	Ref. [46]
*Feresa attenuata*	Pygmy killer whale	JF289171	This study
*Feresa attenuata*	Pygmy killer whale	JF289172	This study
*Peponocephala electra*	Melon-headed whale	JF289175	This study
*Peponocephala electra*	Melon-headed whale	JF289176	This study
*Globicephala macrorhynchus*	Short-finned pilot whale	HM060334	Ref. [17]
Globicephala macrorhynchus*	Short-finned pilot whale	JF339975	This study
Globicephala macrorhynchus*	Short-finned pilot whale	JF339976	This study
*Globicephala macrorhynchus*	Short-finned pilot whale	JF339974	This study
Globicephala melas*	Long-finned pilot whale	JF339973	This study
*Globicephala melas*	Long-finned pilot whale	HM060333	Ref. [17]
*Globicephala melas*	Long-finned pilot whale	JF339972	This study
*Pseudorca crassidens*	False killer whale	JF289173	This study
*Pseudorca crassidens*	False killer whale	JF289174	This study
*Pseudorca crassidens*	False killer whale	HM060332	Ref. [17]
*Grampus griseus*	Risso's dolphin	EU557095	Ref. [28]
*Orcaella brevirostris*	Irrawaddy dolphin	JF289177	This study
Orcaella heinsohni*	Australian snubfin dolphin	JF339978	This study
Orcaella heinsohni*	Australian snubfin dolphin	JF339979	This study
Orcaella heinsohni*	Australian snubfin dolphin	JF339980	This study
Orcaella heinsohni*	Australian snubfin dolphin	JF339981	This study
*Orcaella heinsohni*	Australian snubfin dolphin	JF339977	This study
*Orcinus orca*	Killer whale	GU187186	Ref. [17]
*Orcinus orca*	Killer whale	GU187180	Ref. [17]
Steno bredanensis*	Rough-toothed dolphin	JF339982	This study
*Stenella attenuata*	Pantropical spotted dolphin	EU557096	Ref. [28]
*Sousa chinensis*	Indopacific humpbacked dolphin	EU557091	Ref. [28]
*Tursiops truncatus*	Common bottlenose dolphin	EU557093	Ref. [28]
*Stenella coeruleoalba*	Striped dolphin	EU557097	Ref. [28]
*Delphinus capensis*	Long-beaked common dolphin	EU557094	Ref. [28]
*Tursiops aduncus*	Indian Ocean bottlenose dolphin	EU557092	Ref. [28]
*Monodon monoceros*	Narwhal	AJ554062	Ref. [46]
*Phocoena phocoena*	Harbor porpoise	AJ554063	Ref. [46]
*Inia geoffrensis*	Amazon river dolphin	AJ554059	Ref. [46]
*Lipotes vexillifer*	Yangtze river dolphin	AY789529	Ref. [47]

Asterisks indicate incomplete sequences (see Additional file 1).

### Phylogenetic analyses

Phylogenetic estimates were consistent across Bayesian and maximum-likelihood methods used in this study, including partitioned and unpartitioned analyses on the full data set, and a subset consisting of a single representative per species. With one exception we were able to reject alternative topologies (Table 2). Based on Bayes factors, we found strong support for allowing different partitions of the mitogenome alignment to have distinct evolutionary models in the phylogenetic analysis (see Additional file 2). Although the harmonic-mean estimators of Bayes factors such as those implemented in the software packages used here can be unreliable [30,31], the magnitude of our estimated Bayes factors (see Additional file 2) make it unlikely that we have been misled into selecting a suboptimal partitioning strategy for this alignment. In the partitioned analysis, the mean substitution rate across the 12 protein-coding genes and 2 rRNA genes was 9.86 × 10^-3 ^substitutions/site/My, with a 95% highest posterior density (HPD) interval of 8.45 × 10^-3 ^- 1.13 × 10^-2 ^substitutions/site/My. The coefficient of variations of rates was 0.527 (95% HPD interval 0.405 - 0.648), indicating the presence of substantial rate variation among lineages. The fastest data partition (third codon sites) evolved at about 16.6 times the rate of the slowest data partition (second codon sites).

**Table 2:** The p-values of the approximately unbiased test and weighted or unweighted Kishino-Hasegawa and Shimodaira-Hasegawa tests are provided as well as the bootstrap and Bayesian posterior probabilities of the selected topology.

Test	rank	item	obs	au	np	bp	pp	kh	sh	wkh	wsh
A.	1	A1	-119.3	1.000	1.000	1.000	1.000	1.000	1.000	1.000	1.000
	2	A3	119.3	<0.001	<0.001	0	<0.0001	0	0	0	0
	3	A2	121.3	<0.001	<0.001	0	<0.0001	0	0	0	0

B.	1	B1	-5.0	0.902	0.882	0.883	0.993	0.876	0.926	0.876	0.974
	2	B3	5	0.01	0.01	0.117	0.007	0.124	0.501	0.124	0.237
	3	B2	178.4	<0.001	<0.001	0	<0.001	0	0	0	0

C.	1	C1	-554.2	1.000	1.000	1.000	1.000	1.000	1.000	1.000	1.000
	2	C2	554.2	<0.001	<0.001	0	<0.001	0	0	0	0

D.	1	D2	-291.8	1.000	1.000	1.000	1.000	1.000	1.000	1.000	1.000
	2	D1	291.8	<0.001	<0.001	0	<0.001	0	0	0	0

**Figure 2:**
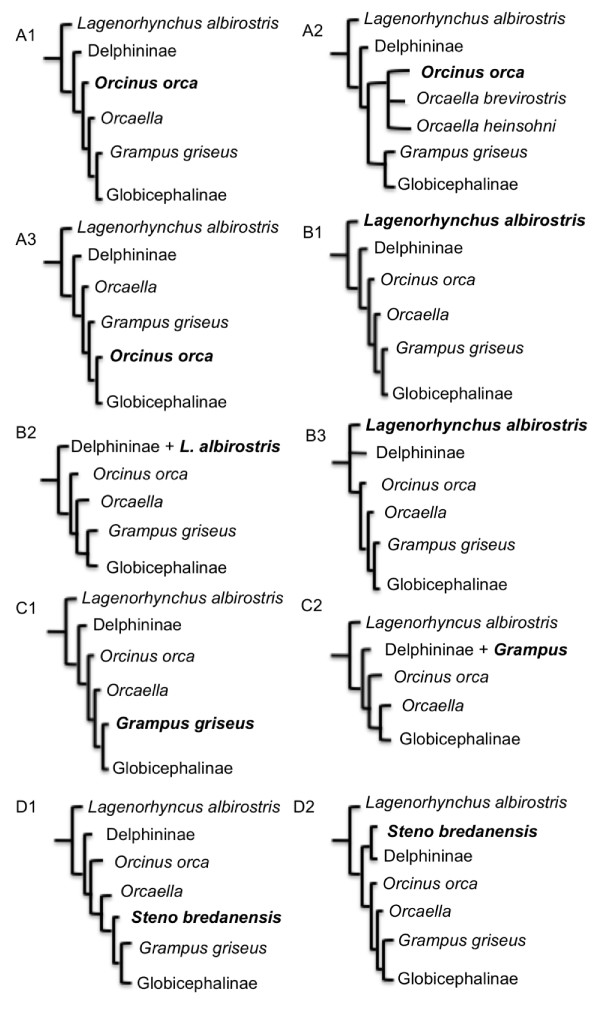
**The alternative topologies tested (Table 2)**. The letters in the alphanumeric label indicates the taxon whose position was tested (also in bold): A = *Orcinus orca*; B = *Lagenorhynchus albirostris*; C = *Grampus griseus*; D = *Steno bredanensis*, while the numbers correspond to item numbers in Table 2.

Tested alternative topologies are shown in Figure 2. Obs = observed log-likelihood difference to the best topology; au = approximately unbiased; np = bootstrap probability of the topology (i.e., the probability that the given topology has the largest likelihood in 10 scaled sets of 10,000 bootstrap replicates); bp = np with 10 non-scaled sets of 10,000 bootstrap replicates; pp = Bayesian posterior probabilities of the model; kh = Kishino-Hasegawa; sh = Shimodeira-Hasegawa; wkh = weighted KH; wsh = weighted SH.

Our analyses strongly support the placement by LeDuc *et al*. [21] of *Grampus griseus *within the Globicephalinae subfamily, a result that now finds wide support from a range of different markers [24,26,27]. In contrast to LeDuc *et al*.'s [21] revisions we were able to reject the suggested grouping of *Orcinus orca *with *Orcaella*: the proposed Orcininae subfamily. Instead we find that *O. orca *is sister taxon to the rest of the Globicephalinae subfamily, which also contains the *Orcaella *genus. We were also able to reject the placement of *Steno bredanensis *in Globicephalinae as proposed by Caballero *et al*. [24] and supported by McGowen *et al*. [26]. Traditionally, and based on morphology alone, *S. bredanensis *has been placed in the subfamily Stenoninae with the genera *Sousa *and *Sotalia *[29]. *Sousa chinensis *has since been moved to the Delphininae subfamily and our analyses suggest that *S. bredanensis *is sister taxon to the rest of the species within this clade. Membership of these subfamilies appears to be supported by shared derived morphological characteristics, however, a thorough cladistic morphological analysis at the genus level remains lacking.

Our analyses suggest *Lagenorhynchus albirostris *and the Delphinidae family are sister taxa, but neither approximately unbiased nor other topological tests (weighted or unweighted Kishino-Hasegawa and Shimodaira-Hasegawa tests) were able to reject an alternative topology (Table 2). However, maximum-likelihood bootstrap values and Bayesian posterior probabilities supported *L. albirostris *as the sister taxon of the Delphinidae family (88.3% and 0.993 respectively), leaving only marginal support for the two alternative topologies tested (Figure 2). The positioning of *L. albirostris *and the three major Delphinidae subfamilies, Globicephalinae, Delphininae, and Lissodelphininae (not represented in our analyses), has been inconsistent among published studies [e.g. [21,22,24,26,27]]. Such phylogenetic uncertainty suggests that this may represent a true species polytomy. However, unlike previous studies, our positioning of *L. albirostris *had high support (Figure 1), and the support of approximately unbiased tests for an alternative topology was only marginally significant (Table 2). Periods of rapid environmental change can lead to rapid or even simultaneous speciation events, which would result in a hard polytomy [12]. Our time-calibrated mitogenomic phylogeny placed the splitting of the three major subfamilies and *L. albirostris *during the late Miocene (11.6-5.3 Mya; Figure 1), a period of fluctuating temperature and sea level [32]. This dating is consistent with the period during which Steeman *et al*. [27] detected a significant increase in net diversification rates within Delphinidae. Therefore, we suggest that a rapid radiation during the period of extreme environmental fluctuations in the late Miocene best explains the lower support for the phylogenetic positioning of *L. albirostris *within Delphinidae.

Consistent with geographical distribution, morphological differences, and a high number of fixed differences in the mtDNA control region [33], we found a deep phylogenetic divergence between *Orcaella brevirostris *and *O. heinsohni *(Figure 1). This divergence is dated to the Pliocene (5.3-2.6 Mya; Figure 1), a period characterised by elevated cyclical fluctuations in sea temperatures and sea levels with an overall trend of cooling temperatures and an increasing west-to-east sea surface temperature (SST) difference across the equatorial Pacific [34-37]. Our data also indicate a rapid radiation of the other extant species within the Globicephalinae subfamily during this period of climatic variation.

### Caveats and recommended future work

The inclusion of nuDNA loci in some of the recent studies [24,26,27] may explain the incongruencies noted above. The mitogenome is a single, maternally inherited, haploid locus and might not wholly reflect the underlying pattern of population divergence and lineage formation (e.g., [38]). Nuclear genes can have greater resolving power for deep-level phylogenetic inference [39] and, when combined with whole mitogenomes, they have also been able to improve resolution in cases of apparent incomplete lineage sorting due to rapid radiations [40]. Historical hybridization is another potential source of incongruence between mtDNA and nuDNA trees [2,12]. This is a particularly important factor to consider if the Biological Species Concept rather than the Phylogenetic Species Concept is being used to define species boundaries, as reproductive isolation is a key criterion for species status under the former [41].

A recent study based on the control regions of the two pilot whale species, *Globicephala melas *and *G. macrorhynchus*, found no support for reciprocal monophyly [42]. It was suggested that this could be due to incomplete lineage sorting or a lack of sufficient data. We found complete lineage sorting with high monophyletic support for all species for which we had multiple representatives, including the two pilot whale species (Figure 1). However, we had small sample numbers from a limited geographical range for all taxa. Including single or low numbers of representatives of each species assumes that the individuals sequenced and included in the phylogenetic analyses are representative of a reciprocally monophyletic clade [41]. However, increased sampling may reveal instances of paraphyly or polyphyly [41]. Increased sampling and mitogenome sequencing, such as conducted on the killer whale [17], is needed to confirm complete lineage sorting for the remaining species included in this study. Increased taxon sampling, such as the inclusion of the species within the Lissodelphininae, may also further reduce phylogenetic error [7,43,44].

## Conclusions

We have used complete mtDNA genome sequences to resolve the phylogenetic relationships within the Delphinidae with high support. Future studies could apply similar methods to resolve para- and polyphyletic genera, e.g., the four *Lagenorhynchus *species within the subfamily Lissodelphininae [45]. Our study further supports previous empirical and theoretical studies [3-7], that increasing sequence data can improve phylogenetic resolution, even in species known to have undergone rapid radiation in the recent past, and can help to discriminate between hard and soft polytomies. However, some clades will remain unresolved, most likely due to simultaneous speciation events [12]. With current high-throughput sequencing techniques, animal mitogenomes can be sequenced relatively quickly and at low cost, and are an attractive candidate for future phylogenetic analyses, particularly if combined with current analytical tools that can aid in the reconstruction of challenging phylogenies.

## Methods

### Sample collection, DNA extraction, amplification and sequencing

Epidermal tissue was obtained by remote biopsying of free-ranging animals [46] and stored in 20% dimethyl sulphoxide (DMSO) saturated with salt [47].

Genomic DNA was extracted from epidermis using the Qiagen DNeasy (Qiagen DNeasy, Valencia, CA, USA) kit following the manufacturer's guidelines. The mitochondrial genome was amplified in 3-5 overlapping amplicons (dependent on DNA preservation) using previously published long-range PCR primers [17] and primers designed specifically for this study (see Additional file 3). Additional sequencing primers were also designed for gap filling using conventional Sanger sequencing at the commercial service offered by Macrogen (Seoul, South Korea). Each 25 μl PCR contained 1 μl extracted DNA, 1× PCR buffer, 2 mM MgSO_4_, 0.4 μM of each primer, 100 nM mixed dNTPs and 0.1 μl High Fidelity Platinum Taq (Invitrogen, Carlsbad, California). PCR amplifications were performed using an MJ Thermocycler with a 4 minute activation step at 94°C, followed by 35 cycles of 94°C for 30 seconds, 62°C for 30 seconds, 68°C for 6 minutes 30 seconds, followed by a final extension period of 72°C for 7 minutes. For amplifications under 6 kb in size the extension time was reduced to 3-5 minutes.

The amplified PCR products were purified using an Invitek PCRapace purification kit (PCRapace, Invitek, Berlin, Germany) and quantified using a NanoDrop spectrophotometer (NanoDrop Products, Wilmington, DE) to determine DNA concentration used to balance and pool amplicons in equimolar ratios. Length of fragment, ratio of fragment lengths per individual, and DNA concentration was taken into account when balancing the samples. Samples were either individually tagged according to Meyer *et al*. [16] and built into shotgun sequencing libraries following the manufacturer's instructions (454 Life Sciences, Branford, CT), or grouped into sets of 8-10, where within each sample set individual libraries were made to contain a different 10 bp multiplexing identifier (MID) tag, allowing libraries to be combined prior to emulsion PCR. Sequencing libraries were quantified by qPCR [16] and pooled at equimolar concentrations. Library pools were divided among regions on GS FLX sequencing runs, using either LR70 or Titanium chemistry (454 Life Sciences). Sequencing data was parsed into individual extractions and identifier tags were removed using a custom tag-removal Perl script (M. Rasmussen, unpublished, University of Copenhagen).

### Phylogenetic analyses

Sequences were assembled using gsMapper (Roche Applied Science, Indianapolis, IN, USA) and aligned by eye using SE-AL v2.0a11 (A. Rambaut, University of Oxford), while Geneious (Biomatters Ltd., Auckland, New Zealand) and Sequencher v4.8 (Genes Code Corporation, Ann Arbor, MI) were used to check coverage and sequence reliability. Conspicuous indels, base position differences, and differences in homopolymeric regions were double-checked and sequences with higher coverage were generally given preference. In order to ensure that the data was not affected by the erroneous incorporation of nuclear pseudogenes (numts) we visually assessed the recovered sequences for the presence of stop codons or frame-shift mutations in the aligned protein-coding genes. We observed no evidence that numts might be present in the data. This may be explained by a combination of (a) the general difficulty with PCR amplifying long amplicons, requiring relatively high levels of template for successful amplication, and thus (b) the fact that mtDNA template copy numbers are much higher than those for nuDNA templates, leading to preferential mtDNA over nuDNA amplification.

A total of 35 mitogenome sequences were used in the analyses, representing 21 species (Table 1). Of these, 18 mitogenomes were amplified and sequenced for this study and 17 mitogenomes attained from Genbank [17,30,48,49], which included 4 outgroup sequences (narwhal, *Monodon monoceros*; harbor porpoise, *Phocoena phocoena*; Yangtze river dolphin, *Lipotes vexillifer*; Amazon river dolphin, *Inia geoffrensis*). The 18 generated sequences consist of 5 species (pygmy killer whale, *Feresa attenuata*; melon-headed whale, *Peponocephala electra*; Irrawaddy dolphin, *Orcaella brevirostris*; Australian snubfin dolphin, *Orcaella heinsohni*; rough-toothed dolphin, *Steno bredanensis*) whose mitogenomes had not been previously sequenced prior to this study.

A single representative mitogenome from each of the 21 species was used for the initial Bayesian phylogenetic analysis and divergence date estimation. This step was taken so that a speciation prior could be used for the tree topology and node times. Sequences were aligned and the 2 rRNA and 12 protein-encoding genes (excluding ND6) were used to form a data set comprising 13,958 sites. Stop codons were removed from all genes and the control region was excluded from analysis due to saturation, repetitive sequences, and alignment ambiguities. The resulting alignment was divided into four partitions: first, second and third codon sites of the protein-coding genes (3,792 bp per partition), and rRNA genes (2,582 bp). A comparison of Bayesian information criterion values in Modelgenerator [50] were used to find the optimal time-reversible substitution model for each partition. This criterion has been found to perform well in relation to other criteria used in evolutionary model selection [51]. The selected models were GTR+I+G for first and third codon sites, HKY+I+G for second codon sites, and TN93+I+G for the rRNA partition. In all cases, rate variation among sites was modelled using a gamma distribution with six categories [52]. There was little variation in the base frequencies across taxa (see Additional file 4).

The Bayesian phylogenetic analysis was performed using BEAST v1.6 [53]. An uncorrelated lognormal relaxed-clock model was used to allow rate variation among branches [54]. A Bayes-factor analysis indicated that this model received decisive support in comparison to a strict-clock model. The four data partitions shared the same relaxed clock but were allowed to have different relative rates. An exponential prior with a mean of 1/3 was used for the standard deviation of the lognormal distribution of rates, and a Yule prior was specified for the tree topology and relative divergence times. To enable the estimation of absolute divergence times in the tree, four calibrations based primarily on fossil calibrations, along with estimated divergence dates from published studies [27,28,55] (see Additional file 5), were incorporated into the analysis. The calibrations were implemented in the form of uniform prior distributions for the ages of the four nodes, and monophyly was enforced on the clades defined by these four nodes.

Posterior distributions of parameters, including the tree topology and divergence times, were estimated by Markov chain Monte Carlo (MCMC) sampling. Samples were drawn every 5,000 MCMC steps over a total of 50,000,000 steps. The first 10% of samples were discarded as burn-in. Convergence to the stationary distribution and acceptable mixing were investigated using the diagnostic software Tracer v1.5 (Rambaut and Drummond, 2007, University of Oxford). From the set of posterior samples, the tree with the highest product of clade credibilities was identified and the branch lengths were rescaled to match mean posterior estimates.

Additional phylogenetic analyses were performed in order to examine the effect of data partitioning. First, the analysis was repeated without data partitioning, so that the protein-coding genes and the rRNA genes shared the same substitution model and mean evolutionary rate. Second, analyses were performed on three data sets in which sites were randomly assigned to the four data partitions mentioned earlier. Randomisation of sites (sampling without replacement) was performed using the Java application SiteSampler v1.1 [56] Support for the different partitioning schemes was examined by assessing Bayes factors, calculated using a harmonic-mean estimator in the software Tracer v1.6 [57,58].

A second set of phylogenetic analyses was performed on the full dataset (35 mitogenomes), including multiple representatives per species, coding missing data as N and using data partitioning for each codon position, rRNA, and control region. For each partition, the best model of molecular evolution that was compatible with models implemented in MrBayes 3.12 [59] was selected using the Bayesian information criterion. The selected models were TN93+I+G for the first and second codon sites, HKY+I+G for third codon sites and the control region, and HKY+I+G for the rRNA genes. Similar models were recovered after RY-recoding a hypervariable part of the control region (nucleotides 15556-15588 according to the *Globicephala macrorhynchus*, Genbank accession number HM060334, reference mitogenome). Bayesian phylogenetic analysis was performed using the 5-partition datasets (with and without RY-recoding of the control region) using MrBayes 3.12 [59]. Posterior distributions of parameters were estimated using two independent MCMC analyses, each comprising one cold and three heated chains. Samples from the posterior were drawn every 1,000 steps over a total of 10 million steps, which appeared to be sufficient to keep the average standard deviation of split frequencies below the critical value of 0.01. The first 25% of samples were discarded as burn-in. A majority-rule consensus tree was constructed from the posterior sample of trees. A supplemental set of analyses was performed with MrBayes 3.12 and PhyML 3.0 [60] using an unpartitioned dataset under a GTR+I+G model as selected using ModelTest [61] and Akaike Information Criterion, with and without RY-recoding of the hypervariable segment of the control region. All analyses yielded identical tree topologies and similar node support values. For maximum-likelihood analyses, the strength of the phylogenetic signal was assessed via non-parametric bootstrapping with 250 pseudo-replicates. In addition, using Consel 0.1 k [62] and site-wise likelihood values recovered from PhyML analyses, levels of statistical support for alternative topologies were evaluated from the p-values of approximately unbiased tests, weighted or unweighted unilateral Kishino-Hasegawa and Shimodeira-Hasegawa tests, and bootstrap and Bayesian posterior probabilities for the selected topologies (Table 2). All trees were drawn using Dendroscope [63].

## Authors' contributions

ADF, EW, JTV, LO, MTPG, PAM, SYWH conceived of the study, and participated in its design and coordination and drafted the manuscript. PAM, DK, MK, GJP, KMR, RS, PV provided samples and carried out DNA extraction. JTV carried out the molecular genetic studies and the sequence alignment. JTV, LO, SYWH performed the statistical analyses. All authors read and approved the final manuscript.

## Supplementary Material

Additional file 1 **Table showing number of sites used for analysis post-partitioning of the 8 incomplete mitogenome sequences**. The complete mitogenome was not sequenced for eight of our samples and therefore only partial mitogenomes were used in the analyses for these eight samples. The exact lengths of these sequences and the number of sites used in the analyses are given in this table. Click here for file

Additional file 2 **Bayes factor statistics of the tested partitioning schemes. **Partitioning schemes tested were unpartitioned, biologically-informed partitioned and randomly partitioned. For the randomly partitioned data sets, the sizes of the four partitions were the same as those in the biologically-informed partitions. Click here for file

Additional file 3 **Table of primers used in this study.** The amplicon of the three primer sets designed by Morin *et al.* 2010 were in some difficult cases split into two, and two new primer sets were designed to amplify shorter sequences. This table contains all primer sequences used to amplify the mitogenome of samples used in this study, including primer melting temperature and the position in the mitogenome of the amplicons the primer set amplifies. Click here for file

Additional file 4 Nucleotide frequencies for concatenated protein-coding genes, rRNA genes, and control region. Nucleotide frequencies for concatenated genes and control region of each amplified mitogenome, including number of sites and means. Taxon names have in most cases been shortened to the first three letters of the genus name followed by the first three letters of the species name, and the sequences amplified in our lab is also followed by sample name. Click here for file

Additional file 5 **Fossil dates used for calibration of divergence times in BEAST**. Dates used to calibrate divergence times in BEAST analysis including their reference. The divergence of *Grampus griseus *from the other Globicephalinae is not based on fossil evidence but on an estimate from Xiong *et al*.'s (2009) analyses. Click here for file
