# Supplementary material for: Mitogenomic phylogenetic analyses of the Delphinidae with an emphasis on the Globicephalinae
Source: BMC Evol Biol. 2011 Mar 10;11:65. doi: 10.1186/1471-2148-11-65 (PMC3065423; doi:10.1186/1471-2148-11-65)
Supplement: Additional file 1 — Table showing number of sites used for analysis post-partitioning of the 8 incomplete mitogenome sequences. The complete mitogenome was not sequenced for eight of our samples and therefore only partial mitogenomes were used in the analyses for these eight samples. The exact lengths of these sequences and the number of sites used in the analyses are given in this table. [file 1471-2148-11-65-S1.PDF]

Additional file 1: Table showing number of sites used for analysis post-partitioning* of the 8 incomplete mitogenome sequences.

Sample	Total sequenced	Missing sequence	Used in analysis*
GlomelG4_2	10,681	5,707	9,540
GlomacG1	13,488	2,900	12,249
GlomacG3	13,908	2,480	12,669
Orchei08	14,072	2,317	12,728
Orchei22	14,090	2,299	12,729
Orchei02	14,068	2,321	12,729
Orchei06	14,070	2,319	12,730
StebreS9	16,272	113	16,266
