# Supplementary material for: Mitogenomic phylogenetic analyses of the Delphinidae with an emphasis on the Globicephalinae
Source: BMC Evol Biol. 2011 Mar 10;11:65. doi: 10.1186/1471-2148-11-65 (PMC3065423; doi:10.1186/1471-2148-11-65)
Supplement: Additional file 2 — Bayes factor statistics of the tested partitioning schemes. Partitioning schemes tested were unpartitioned, biologically-informed partitioned and randomly partitioned. For the randomly partitioned data sets, the sizes of the four partitions were the same as those in the biologically-informed partitions. [file 1471-2148-11-65-S2.PDF]

Additional file 2: Bayes factor statistics comparing the use of unpartitioned, biologically-informed partitioned, and randomly partitioned data sets. For the randomly partitioned data sets, the sizes of the four partitions were the same as those in the biologically-informed partitions.

Partitioning	Harmonic mean log-likelihood	Log_{10} Bayes factor*
4 biologically-informed partitions<br>(1st, 2nd, and 3rd codon sites, rRNA genes)	-64,036.82	-
4 random partitions - replicate 1	-65,155.71	-486.36
4 random partitions - replicate 2	-65,156.41	-486.67
4 random partitions - replicate 2	-65,155.57	-486.3
Unpartitioned	-67,689.84	-1,586.92

*Log_{10} Bayes factor in relation to the biological partitioning scheme.
