# Supplementary material for: Mitogenomic phylogenetic analyses of the Delphinidae with an emphasis on the Globicephalinae
Source: BMC Evol Biol. 2011 Mar 10;11:65. doi: 10.1186/1471-2148-11-65 (PMC3065423; doi:10.1186/1471-2148-11-65)
Supplement: Additional file 3 — Table of primers used in this study. The amplicon of the three primer sets designed by Morin et al. 2010 were in some difficult cases split into two, and two new primer sets were designed to amplify shorter sequences. This table contains all primer sequences used to amplify the mitogenome of samples used in this study, including primer melting temperature and the position in the mitogenome of the amplicons the primer set amplifies. [file 1471-2148-11-65-S3.PDF]

Additional file 3: Table of primers used in this study. The amplicon of the three primer sets designed by Morin *et al.* 2010 were in some difficult cases split into two and two new primer sets designed to amplify shorter sequences.

Name	Primer sequence	Amplicon	Tm °C	Reference
LR2F	5'-AACTGCTAATTCATGTGCTCCA-3'	11,716-2,723	52.0	Ref. 17
LR2R	5'-TAAAAGTTTAAGTTTTATGCAATTGCCA-3'		56.0	Ref. 17
LR2F1	5'-GCTCCATACTTAACAATGTGGCT-3'	11,695-16,153	55.5	This study
LR2R1	5'-CACCTGTTGGTTGTGCTAGT-3'		55.3	This study
LR2F2	5'-CCTTACATCGGCACTACCCT-3'	14,656-2,315	56.4	This study
LR2R2	5'-GTTTTAATCACTCGGAGGGT-3'		52.4	This study
LR3F	5'-CTTGTATGAATGGCCACACG-3'	2,114-6,937	51.0	Ref. 17
LR3R	5'-TAGAGGGGGTTCGATTCCTT-3'		52.0	Ref. 17
LR3F1	5'-GGACTTGTATGAATGGCCACACGAGGG-3'	2,091-4,731	66.0	This study
LR3R1	5'-GACACTCACCTCCCTCAGGAGG-3'		65.0	This study
LR3F2	5'-CCGTACAATCCAACCTTCACCTTACTAAACCTG-3'	4,539-6,962	65.0	This study
LR3R2	5'-CCGATTAGAGGGGGTTCGATTCCTTCC-3'		66.0	This study
LR4F	5'-CCTCCACCATACCACACATTC-3'	6,898-11,800	52.0	Ref. 17
LR4R	5'-TGTCAGTAGGGTGGAAGAGG-3'		49.0	Ref. 17
LR4F1	5'-CGGATGTCCTCCACCATACCACACATTC-3'	6,898-9,310	62.6	This study
LR4R1	5'-GGCGTATGAAGCAGATAATGAGG-3'		54.9	This study
LR4F2	5'-GCCCCATTTACAATCTCAGACGG-3'	9,242-12,004	58.3	This study
LR4R2	5'-GGATAGTGGTTCAGTGTCAG-3'		52.7	This study
LR4F1.1	5'-GGAAGGAATCGAACCCCCTC-3'		57.9	This study
LR4R1.1	5'-GCCATAGATTCCGTCTGAGA-3'	6,921-9,236	53.8	This study
