# Supplementary material for: Mitogenomic phylogenetic analyses of the Delphinidae with an emphasis on the Globicephalinae
Source: BMC Evol Biol. 2011 Mar 10;11:65. doi: 10.1186/1471-2148-11-65 (PMC3065423; doi:10.1186/1471-2148-11-65)
Supplement: Additional file 4 — Nucleotide frequencies for concatenated protein-coding genes, rRNA genes, and control region. Nucleotide frequencies for concatenated genes and control region of each amplified mitogenome, including number of sites and means. Taxon names have in most cases been shortened to the first three letters of the genus name followed by the first three letters of the species name, and the sequences amplified in our lab is also followed by sample name. [file 1471-2148-11-65-S4.PDF]

Additional file 4: Nucleotide frequencies for concatenated protein-coding genes, rRNA genes, and control region.

Taxon	A	C	G	T	No. of sites
Delcap	0.328	0.263	0.124	0.284	14874
Feratt35	0.329	0.270	0.123	0.278	14876
Feratt36	0.329	0.270	0.123	0.278	14876
Glomac52	0.328	0.267	0.124	0.281	14876
Glomac65	0.328	0.267	0.124	0.280	14876
GlomacG1	0.323	0.277	0.115	0.285	12221
GlomacG3	0.321	0.276	0.117	0.286	12639
GlomeG4_2	0.318	0.271	0.126	0.286	9508
GlomelG4	0.329	0.267	0.123	0.280	14880
GlomelG5	0.329	0.267	0.123	0.280	14880
Gragri	0.327	0.268	0.126	0.279	14874
I_igeo	0.325	0.287	0.128	0.260	14873
Lagalb	0.328	0.264	0.125	0.283	14877
Lipvex	0.336	0.275	0.120	0.268	14879
Mo_mo_	0.329	0.280	0.124	0.267	14872
Orcbre97	0.328	0.266	0.124	0.282	14875
Orchei02	0.321	0.275	0.118	0.286	12693
Orchei06	0.321	0.274	0.118	0.287	12694
Orchei08	0.321	0.274	0.118	0.287	12695
Orchei22	0.321	0.274	0.118	0.287	12695
Orchei28	0.329	0.265	0.124	0.282	14878
Orcorc49	0.326	0.273	0.125	0.275	14872
Orcorc93	0.326	0.273	0.125	0.275	14872
PepeleM6	0.329	0.271	0.123	0.277	14878
PepeleP5	0.329	0.271	0.123	0.277	14878
Phopho	0.318	0.275	0.133	0.274	14870
Psecra92	0.331	0.268	0.122	0.279	14881
Psecra96	0.331	0.268	0.122	0.279	14877
PsecraP4	0.331	0.268	0.122	0.279	14879
Souchi	0.329	0.266	0.125	0.281	14872
Steatt	0.328	0.265	0.124	0.283	14873
StebreS9	0.329	0.267	0.124	0.280	14877
Stecoe	0.328	0.265	0.124	0.282	14872
Turadu	0.327	0.264	0.126	0.283	14873
Turtru	0.327	0.266	0.125	0.282	14875
Mean	0.327	0.270	0.123	0.280	14333
