# Supplementary material for: Mitogenomic phylogenetic analyses of the Delphinidae with an emphasis on the Globicephalinae
Source: BMC Evol Biol. 2011 Mar 10;11:65. doi: 10.1186/1471-2148-11-65 (PMC3065423; doi:10.1186/1471-2148-11-65)
Supplement: Additional file 5 — Fossil dates used for calibration of divergence times in BEAST. Dates used to calibrate divergence times in BEAST analysis including their reference. The divergence of Grampus griseus from the other Globicephalinae is not based on fossil evidence but on an estimate from Xiong et al.'s (2009) analyses. [file 1471-2148-11-65-S5.PDF]

Additional file 5: Fossil dates used for calibration of divergence times in BEAST. The divergence of *Grampus griseus* from the other Globicephalinae is not based on fossil evidence but on an estimate from Xiong *et al.*'s (2009) analyses.

Divergence events	Min-Max age (Mya)	Reference
Inia and *Lipotes*	18.7-22.7	Steeman *et al*. 2009
Grampus and Globicephalinae	5.2-9.2	Xiong *et al.* 2009
G. melas and *G. macrorhynchus*	2.2-4.1	Aguirre-Fernandez *et al.* 2009
Phocoena and *Monodon*	9.8-11.2	Xiong *et al*. 2009
